# Experiential Value of Technologies: A Qualitative Study with Older Adults

**DOI:** 10.3390/ijerph19042235

**Published:** 2022-02-16

**Authors:** Shital Desai, Colleen McGrath, Heather McNeil, Heidi Sveistrup, Josephine McMurray, Arlene Astell

**Affiliations:** 1 Social and Technological Systems Lab, School of Arts Media Performance and Design, York University, Toronto, ON M3J 1P3, Canada; 2 School of Occupational Therapy, Western University, London, ON N6A 3K7, Canada; cmcgrat2@uwo.ca; 3 SE Research Centre, SE Health, Markham, ON L3R 6H3, Canada; heathermcneil@sehc.com; 4 School of Rehabilitation Services, Faculty of Health Sciences, University of Ottawa, Ottawa, ON K1N 6N5, Canada; hsveist@uottawa.ca; 5 Lazaridis School of Business & Economics, Wilfrid Laurier University, Waterloo, ON N2L 3C7, Canada; jmcmurray@wlu.ca; 6 Department of Occupational Sciences & Occupational Therapy, University of Toronto, Toronto, ON M5G 1V7, Canada; arlene.astell@uhn.ca; 7 Department of Psychiatry, University of Toronto, Toronto, ON M5G 1V7, Canada; 8 KITE, Toronto Rehabilitation Institute, Toronto, ON M5G 2A2, Canada

**Keywords:** experiential value, technology, co-creation, aging well, user-centred design

## Abstract

This study investigated the experiences of older adults with technologies they own and determined how they value them. Thirty-seven older adults participated in a Show and Tell co-creation session at a one-day workshop. Participants described why they loved or abandoned technologies they own. Their responses were recorded and analysed using Atlas.ti 22.0.0. Seven main themes representing experiential value in older adults emerged from the analysis: Convenience, Economy, Learning and Support, Currency of Technology, Privacy and Security, Emotions and Identity aspects of their experiences. This qualitative study has resulted in implications to design that recommends (a) Design for product ecosystems with technologies and services well-coordinated and synchronized to facilitate use of the technology (b) Create awareness and information on privacy and security issues and technical language associated with it (c) Make anti-virus and anti-phishing software accessible to older population (d) Design technologies as tools that allow older adults to identify themselves in the community and family (e) Create services that make technologies and services in the ecosystem affordable for the older adults. The outcomes of this study are significant as they provide recommendations that target systemic issues which present barriers in the use of technology.

## 1. Introduction

We are in the middle of a longevity revolution. According to World Population Prospects 2019, one in six people in the world will be over the age of 65 by 2050. On average, people aged 65 in 2015–2020 will live up to 82 years, which is expected to increase to 84 years by 2045–2050. Seniors in Canada will number over 9.5 million and represent 23 percent of Canadians. The average life expectancy at birth is expected to rise for both men and women in Canada by 2036.

Technologies could support older adults to live independently and age gracefully. Digital tools and services could enable active ageing, tackle social isolation in seniors, and provide access to healthcare, especially during the COVID-19 pandemic [1]. According to the World Economic Forum’s Technology Pioneers and Global Innovators Community [2], technologies can improve caregiver effectiveness, increase accessibility to healthcare, facilitate healthy ageing at home, improve early detection and early intervention for metabolic disease and neuropsychiatric disorders, ensure ethical and responsible use of health data, treat non-communicable disease, predict illness using AI, help older adults stay longer in their homes through simple interfaces that make everyday tasks easy, and scale solutions for faster and smarter pathways. 

In comparison to young people [3,4,5], older adults are slower to adopt new technologies in everyday life. Seniors mostly use technology products to access health care, health management, and banking services. However, their technology use is limited to times of need, for example, during the COVID-19 pandemic. The world is moving towards a technology-driven economy that promises improved and accessible services. According to a McKinsey Global Survey of executives, the COVID-19 pandemic has sped up the adoption of technologies by several years, and these changes will be here to stay for the long haul [6]. There is a growing concern that technologies and services will remain inaccessible to seniors, affecting their quality of life. Thus, it is important to design technology-mediated products to provide improved care and facilitate independent living in older adults [7]. It is essential that these technologies be inclusive and accessible for seniors. However, these goals are only achievable by involving seniors in the design and development process.

Prior studies on technology adoption in older adults have mainly focused on the factors that influence intention to use the technology, decision making, and attitudes towards technology [8,9,10]. Perceived usefulness and usability of the technology were cited as primary barriers to adoption [8]. These studies qualified adoption as the short-term use of a product rather than long-term acceptance in everyday life. The studies also used models such as Technology Adoption Model (TAM) to evaluate the use of computers, communication technologies, and smart home technology in seniors [11,12,13]. However, the application of these models to the context of technology use in older adults is limited. These models were developed in studies either with a limited sample of older adults or none at all.

Prior experience with technologies is a driver of attitudes towards technology. To better understand long-term technology adoption and acceptance in older adults, this study aims to investigate the experiences of older adults with technology that they owned in the past or currently own. This will inform us on how older adults value technologies based on their experiences with everyday technologies.

## 2. Related Work

Experience is a “stream of feelings, thoughts and action; a continuous commentary on our current state of affairs” [14]: it is ubiquitous and unconscious, but it is also something that the person encountering is able to explain and describe [15]. Experience is dynamic and changes as a person engages with a technology or product. The person using a technology goes through a variety of experiences such as emotional, physical, and cognitive, depending on the human systems that the technology affects. These experiences will determine whether a person continues to engage with a product, disengage (by cancelling activities associated with the technology), or reengage [16]. For example, a person could stop exercising after negative incidents with their personal activity tracker. This example explains how Kahnemann’s notion of “continuous commentary” determines our objective happiness [14]. We may enjoy a positive experience with a technology and decide to continue using it. Then, over a period of an interval, an integral of these experiences could contribute to happiness.

The experiences, encounters, and memories that people have with technology products determine a product’s value. Understanding this kind of user value (driven by how people feel about the products after having used them) is essential to develop products that fit in the human value system and drive adoption and acceptance of these products. For example, technologies such as text messaging, emails, and chat messaging allow people to stay in touch; they augment people’s ability to communicate, and at the same time, fit the human value system that believes communication enriches relationships between people [17].

Experiential value is the user’s perception of products and interfaces after they have experienced the product [18]. Sheth, Newman, and Gross attribute experiential value to functional, conditional, social, emotional, and epistemic utility [19]. Value is generated during and after the process of interacting with the interfaces. People could experience emotional reactions, feelings, fun, and playfulness while interacting and using technologies [20,21]. These feelings and sensitizations determine a person’s level of satisfaction and drive decision making [22].

People develop experiential value through the extrinsic and intrinsic benefits that a product offers [23,24]. In the context of technology use, extrinsic benefit derives from the utility of a product, or in other words, how a technology product benefits the user [25]. On the other hand, intrinsic value derives from the “appreciation of an experience for its own sake, apart from any other consequence that may result” ([26], p. 40). Babin, Darden and Griffin note that intrinsic value is based on the subjective and personalized nature of encounters with technology ([27], p. 646).

Conventionally, value has been measured as a trade-off between product quality and price [28,29]. However, older adults’ investment of money, time, and effort in a product goes beyond the monetary value. A feeling of satisfaction resulting from the use of a product is attributed to the subjective feelings and encounters of older adults with technology products. However, identifying these subjective experiences in the context of older adults and technology use requires further investigation. The importance of experiential value is aptly presented by Holbrook, “value resides not in the product purchased, not in the brand chosen, not in the object possessed, but rather in the consumption experience(s) derived there from” [30].

To develop technologies that older adults will value and integrate into their everyday lives, we must understand their experiences with technology products they currently own and have used in the past. This paper describes a study that was carried out to determine how older adults value technology products while considering their direct experiences with a range of everyday technologies. The outcome of this study will enable designers and technology developers to come up with technology products for older adults that enhance seniors’ use of digital tools and services and drive technology adoption. The development of these products is increasingly crucial during the ongoing COVID-19 pandemic (and beyond) as most services have moved to remote and online formats. Marginalization of seniors from digital innovation has affected their access to these services and, thus, their quality of life. The outcomes of this study will help us reduce the digital divide for older adults’ use of technology through people-centric design of technologies.

## 3. Methods

### 3.1. Procedure

The authors conducted a one-day participatory workshop with older adults, caregivers, technology developers, researchers, and community partners at Communitech [31], an innovation hub in Kitchener, Canada. TUNGSTEN (Tools for User Needs Gathering to Support Technology Engagement) is a set of human-centred methods that help researchers involve older adults in the technology development, testing, and implementation processes, from the original conception of ideas to the adoption of products by the users. Show and Tell, one of the TUNGSTEN tools, is an activity designed to understand what makes people love or abandon technology. This tool was used for data collection during this entire study.

Prior to the workshop, participants were asked to bring at least one product they loved and one product they had abandoned. If the technologies were difficult to bring, they were asked to bring a photograph instead. On arrival, participants were allocated to a round table. Each table consisted of at least one older adult, caregiver, technology developer, and healthcare practitioner. If older adults came with a caregiver, they were seated together at the same table as per request.

After introductions, each participant had a turn to show the technology that they loved to the rest of the participants at their table. Participants described the product and why they loved it. They then explained where they got it from; whether it was a gift or a self-purchase; and if it was a gift, who it was from. One participant at each table acted as a scribe and filled in a recording sheet for each item described by the participants. Once all participants described their loved item, the process was repeated for their abandoned item. The scribe also recorded this information on a second recording sheet for abandoned items. Each table then reported their loved and abandoned items to the whole room. Finally, the facilitator summarized the comments from the participants.

Reliability in data collection was ensured by taking precautions against intentional or accidental distortions and biases [32]. The Show and Tell method eliminates power balance issues that exist in other ethnographic methods, such as interviews and focus groups where a researcher leads the session or agenda. The co-creation process refers to an act of collective creativity between two or more people, such as designers, other disciplinary experts, and people who are not trained in design. These individuals work together in the design development process.

The proceedings of the workshop were audio and video recorded for analysis. Step- by-step instructions for the Show and Tell tool are described at http://tungsten-training.com (accessed on 14 October 2021). The recording sheets can be downloaded from the website and used in future workshops on technology and older adults.

### 3.2. Participants

Sixty-eight participants attended a one-day interactive workshop on technology and ageing. Out of these participants, thirty-seven were older adults, and thirty-one were partners/caregivers, technology developers, healthcare practitioners, researchers, and community partners. Belonging to one of these categories was the only criterion to participate in the workshop. Participants were recruited for the workshop session through advertisements/flyers and word of mouth. Flyers were distributed through AGE-WELL email lists and social media platforms. Participants were not known to the researchers prior to the workshop. Participant sample size was decided based on the capacity of the room that was available for the workshop at Communitech. The Show and Tell co-creation method requires an even number of participants at each round table with at least one older adult, caregiver, technology developer, and healthcare practitioner.

### 3.3. Materials

- Video cameras
- Still cameras
- Host and facilitators
- Cabaret style tables and chairs
- Roving microphone
- Recording sheets

### 3.4. Ethical Considerations

Research Ethics Board approval for the study was obtained from the Ontario Shores Centre for Mental Health and Sciences in Canada. Prior to the workshop, the purpose of the study was communicated to all participants, and informed consent was explained verbally. Then, a written informed consent sheet was distributed explaining the study and the ethical considerations, which participants signed and returned. In addition, consent to videotape for the purpose of later analysis was obtained from all participants.

### 3.5. Data Synthesis

The audio video recordings of the data and the recording sheets from the workshop were analyzed using Atlas.ti 22.0.0 (Atlas.ti Scientific Software Development GmbH, Berlin, Germany). In qualitative analysis, the researcher used a phenomenology approach to identify and set aside preconceived ideas, as well as generate emergent themes [33]. The videos were transcribed by a research assistant who was not present at the workshop. Before starting the analysis, the researcher set aside their own preconceived ideas, influenced by past experience and knowledge, to identify themes pertaining to the everyday experience of older adults with technology and determine the experiential value of those technologies. Memos of preconceived bias and notions were created in Atlas.ti [34,35]. The researcher (author 1) watched the audio video recordings of the workshop and read the recording sheets several times to understand the phenomenon in the context of experiences with everyday technologies. The audio video data and recording sheets were coded in Atlas.ti using an inductive approach to identify themes representing the everyday experiences of older adults with technologies they own and determine how they valued those technologies.

Rigour in the determination of heuristics was ensured through detailed observations of all the data. Every code was checked twice and, at times, thrice. Reliability in coding was ensured by carrying out inter-coder analysis (ICA) of the coded data. All coding was done by Author 1. Once a stable coding system was built, another coder (research assistant in the lab) who was not involved with the project and the code system development process coded a subset of the data. Once a satisfactory ICA coefficient was achieved in this process, it assured that the codes could be understood and applied by others. Author 1 continued to use the coding system to finish coding the data.

Krippendorff’s alpha coefficient (α) is used to quantify the extent of agreement between the coders. The coefficient values could range from 0 to 1, where 0 is perfect disagreement, and 1 is perfect agreement. Regarding optimum values for the coefficient, Krippendorff suggests, “[It] is customary to require α ≥ 0.8. Where tentative conclusions are still acceptable, α ≥ 0.667 is the lowest conceivable limit” ([32], p. 241).

ICA was carried out in Atlas.ti to determine the level of agreement between the coding carried out by Author 1 and the coding carried out by the research assistant. Krippendorff’s alpha coefficient (α) was found to be 0.85, and according to Krippendorff [32], the agreement between the two sets of coding was strong. Furthermore, since *p* = 0.000, Krippendorff’s alpha coefficient (α) is statistically significantly different from zero.

## 4. Results

### 4.1. Technologies Loved and Adopted by Older Adults

**Figure 1:**
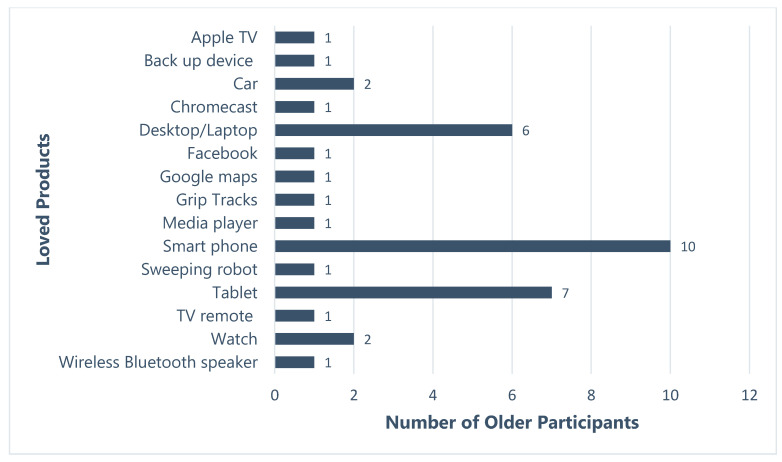
Loved and adopted products (*n* = 37) presented (verbally or physically) by the older adults.

Smartphones were the single most popular technology loved by fourteen of the older adult participants (see Figure 1). To justify this popularity, older adults cited the technology’s portability, multi-functionality, ability to share pictures, and ability to text friends and family.

*“… [have] four children who keep in contact by texting. [have] 2000 pictures of grand kids on my phone…rarely use for phone calls. [I] get phone calls from kids.”* (Participant 18, female)

*“… [I] can talk, text in the car while driving…”* (Participant 12, male)

*“… it is not just a phone but a computer in your hand. Google maps, health monitoring apps, GPS, everything is in it.”* (Participant 07, male)

Six older adults identified their laptops as the technology they loved. Two of these adults owned a Macbook Pro, which according to them, has a greater ability to block viruses and Malware popups than Windows computers. Participants also greatly valued the portability afforded by the laptops.

*“[I] sit on my kitchen table and there I have a computer. I move to the lounge, and I carry it with me. I can skype from anywhere in my house.”* (Participant 15, female)

*“[I watch] online entertainment on my laptop wherever I go. Whether I am at home or traveling, I still can watch all my programs on my laptop.”* (Participant 25, male)

Ten older participants at the workshop loved their tablets, with iPads being the most popular device. Portability and multi-functionality were the driving forces behind the adoption of tablets:

*“[tablets are] light, compact mini-computers.”* (Participant 04, male)

Two older adults mentioned their cars as their loved technology, stating the freedom and utility that their vehicles offer to carry out daily tasks and activities:

*“… [I] love my car. It gives me freedom to do things that I want to do and love to do.”* (Participant 30, male)

Two older adults adopted watches; one participant owned a smartwatch while the other owned a digital watch. Smartwatches allow older people to check the time, make phone calls and track their physical activity. These devices give them access to numerous apps with various applications, meaning that older adults do not have to carry different gadgets for different applications.

*“[I] can do lot more with [my smartwatch] than just seeing time.”* (Participant xx, male)

Older adult who adopted a digital watch did not mention the reason for its adoption.

### 4.2. Technologies Hated and Abandoned by Older Adults

**Figure 2:**
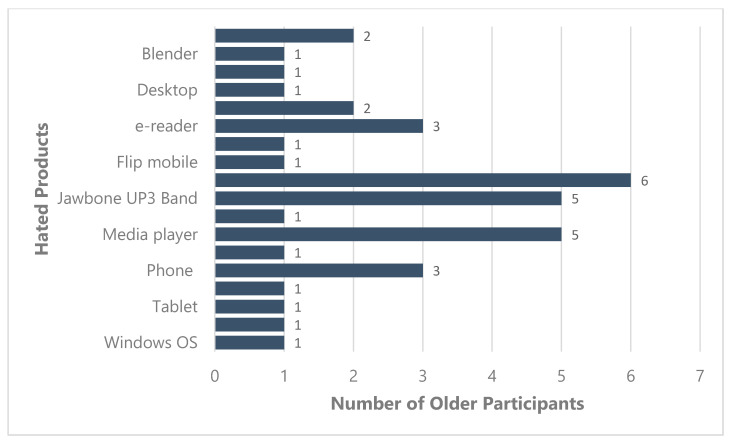
Hated and Abandoned products (*n* = 37) presented by older adults at the workshop.

Desktop computers were rejected by four older adults who stated a lack of portability and desire to use newer technologies (see Figure 2).

*“Desktop computer has no flexibility to use in different locations or to travel.”* (Participant 05, male)

*“… desktop computers are obsolete… I want to use new technologies, laptops, tablets are good to use.”* (Participant 11, female)

One older adult had abandoned her landline and adopted a smartphone. Five older participants had rejected some form of mobile phone. Blackberry was the most abandoned device (three participants). One participant reported she no longer used the flip mobile she owned. Participants cited performance-related issues and a desire to use the latest model smartphone as reasons for rejecting these phones.

*“Blackberry playbook was very slow.”* (Participant 09, male)

*“Younger generations don’t know how to use blackberry. So [I] changed to [another] smartphone.”* (Participant 10, female)

*“Old cell phone was too hard to use. [it] was not up to date.”* (Participant 10, female)

Three older adults had rejected their in-vehicle GPS, citing difficulty understanding its directions. Instead, they had adopted an alternate navigation system on their smartphones, such as Google maps.

*“[I] stopped using my GPS in the car. The instructions were confusing. [I] could not understand what she said. [I] started using Google maps and I am happy with it.”* (Participant 12, male)

Four older adults had rejected a media player (i.e., DVD, cassette player, tape deck, IPod) and instead adopted a smartphone or a tablet to watch movies/programs and listen to music. Portability in a smartphone allows them to enjoy media resources anywhere and anytime.

*“[Television] is expensive and has limited content.”* (Participant 3, female)

*“[Cassette player] was difficult to operate.”* (Participant 32, male)

*“DVD player is no longer in use…because there are new devices out like blue ray or Netflix.”* (Participant 29, male)

### 4.3. Thematic Analysis

**Figure 3:**
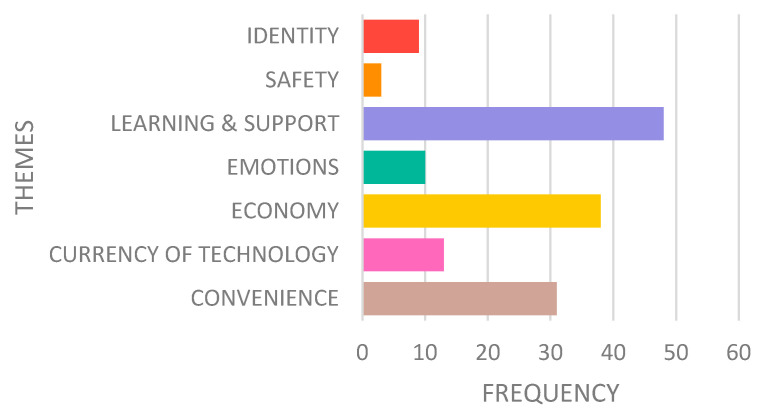
Distribution of the themes that contribute to experiential value in older adults.

Data analysis found seven main themes that represent how older adults value and experience technologies—shown in Table 1 and explained below. Figure 3 shows the distribution of themes (frequency).

#### 4.3.1. Convenience

##### Ease of Use

Older adults value the convenience of using technology for daily tasks. Participants cited how a technology’s functionality makes their tasks easy. Older participants looked to technologies to make everyday activities, such as communicating with family and friends, easy and comfortable. Once the older adults started using the technologies, features such as portability and usability became important to continue using the technology.

*“… [it was too] complicated to download photos with [Kodak Easy Share photo frame].”* (Participant 10, female)

Older participants value the ability of the technologies to adapt its features to their individual specific needs.

*“… nice that you can adjust screen brightness, font size and volume [on iPhone].”* (Participant 12, male)

The convenience of carrying out tasks with a technology however depends on the availability of required resources and infrastructure for the use of the technology.

*“[Smartphone] use is limited to calling and texting due to limited amount of data.”* (Participant 10, female)

Multi-functionality and portability of technologies such as smartphones and laptops afforded convenience to older participants.

*“… [I] use laptop for everything.”* (Participant 32, male)

*“[My IPad] was everything for me when I was in a rehab hospital…communicates with family, email, photos, music, books, filing system, etc.”* (Participant 13, female)

##### Access to Resources

Use of technologies require resources. A lack of access affects older adults’ experience with the technologies. Lack of some resources, such as WIFI, makes some technologies completely unusable while some aspects, such as download cost, makes technologies unaffordable.

*“… [E-readers] have a cost of download which is not affordable.”* (Participant 5, female)

One senior participant could not get technology to work as he could not find instructions on how to link his mobile phone to the Fitbit.

*“[My Fitbit] did not have instructions to link it to the smartphone.”* (Participant 16, male)

#### 4.3.2. Economy

Economic value is represented by the costs incurred in the purchase and the subsequent use of a technology. Older adults assess the economic value of technology products based on the economic benefits offered during the purchase of the product. An additional factor is the cost-effectiveness of the products and the various features they offer. The economic benefits derived from technology products is highly valued by older adults. Older adults value economic benefits such as discounts, installment payments, etc., offered by retail outlets during the purchase of a product. This value was evident in cases where the older participants had purchased the product themselves. Older adults at the workshop expressed their concern over the cost of using technologies, such as internet or data subscription costs associated with smartphone apps, desktop/laptop computers, downloading e-books, etc. This in-use economic value, along with the purchase economic value, decided whether older adults adopted technology products in their everyday lives.

#### 4.3.3. Learning and Support

Learning how to use a new technology device and access to ongoing support is important for older adults. Nonetheless, it is essential that these adults have autonomy, independence, and the ability to proceed at their own pace. As Figure 3 indicates, the theme of learning and support earned the highest frequency of codes. Compared to other themes, older participants most value the learning and support offered by a technology. 

Based on their experiences with technologies, participants identified four crucial forms of learning and support: (1) clear and detailed instructions, (2) ongoing technical support, (3) training, and (4) self-paced learning. The resources to support technology learning will get seniors started when the product is newly purchased and provide ongoing assistance as they adopt and use the technology. At the same time, the product should offer opportunities for older adults to learn and explore by themselves. Older adults want to figure out details about the product by themselves without relying on help from others. Thus, self-paced self-learning is important for independent and self-reliant access to technologies. One older participant did not find the instructions for the Samsung S7 phone that she owned useful. The owner then resorted to online information through Google search.

*“I read the instructions which were not useful. Then I went to Google to find additional information on how to reboot the phone and setup software applications.”* (Participant 24, female)

One older participant reported the adequacy of instruction booklets for Apple products, such as the Macbook Pro and IPad. One older participant reported that his Windows 10 computer had poor user instructions, so he sought help from his family. However, he only started using the computer when he found a book to help him learn how to use it:

*“… [I] could not understand instructions for my Windows 10 computer. I took help from my family. but it was the book titled ‘Windows 10 for seniors’ that I bought to learn how to use the computer helped me get started [using the computer].”* (Participant 23, male)

Some older participants at the workshop had attended training sessions and clubs organized at community centres, malls, library, retail stores, etc., which played a crucial role in learning how to use the technologies. Access to training support enhances experience of older adults with technologies.

*“[I] learned [to use my Android phone] through computer club for seniors.”* (Participant 32, male)

Training support should be cost effective. One participant suggested that the government should pay for educational programs for seniors. Additionally, older adults expressed the need to have access to ongoing technical support from the technology developer or manufacturer.

*“It would be useful to know about or/and have access to a ‘techy person to come to the house to help [with] a technical issue.”* (Participant 36, male)

Most participants reported that they receive help from their family and friends for ongoing technical support because of the unavailability of support from manufacturers or in their communities. One older adult abandoned his Blackberry phone because his children were not familiar with the device and unable to provide ongoing technical support.

#### 4.3.4. Currency of Technology

Older adults desire to use the latest modern technologies. They like to use the latest models and stay up to date with technological innovations. For example, senior participants abandoned technologies such as DVD players, landlines, tape decks, iPods, cars, desktop computers and Jawbone UP3 bands because they were obsolete with newer advanced models and options available in the market.

#### 4.3.5. Privacy and Security

Participants at the workshop expressed the importance of safety and security while using technologies. They expressed concerns over malware and viruses getting into their computers. Older adults were also concerned about sharing private information, such as health-related details and demographic information, as they were unsure with whom the information would be shared.

#### 4.3.6. Emotions

Activities afforded by technologies drive emotions and sentiments in older adults. They derive pleasure from the product aesthetics. Participant 16 abandoned her blender because it looked “ugly.” Similarly, for older adults to adopt wearable technologies in everyday use, the products should look good and suit their personal styles. Seniors also associate pleasure with the experience of fun and playfulness while using technologies. Participant 13 expressed how much she enjoys playing games on her iPad. She stated that her IPad kept her amused while undergoing rehab in the hospital and enabled her to communicate with her loved ones. Older adults also derive pleasure from technological experiences that allow them to share tangible things, such as photographs, and intangible things, such as affection and stories. Some participants stated that they loved their smartphones because the devices allow them to receive calls from their children. Similar experiences were described with laptops. One older participant loved sharing pictures of his grandchildren and great grandchildren on Facebook. Another older participant sent gifts and flowers to his children from his laptop. Another enjoyed keeping in touch with his friends on WhatsApp. He also stated that using the app allowed him to be a grandfather to his grandchildren as he could share stories and music with them. Some participants loved technologies, such as smartphones, computers, and tablets, because they enjoy going through photographs of their children and grandkids.

#### 4.3.7. Identity

Older adults derive identity through their roles in their communities, and they value technologies that facilitate this. Social interactions are relevant to older adults when those interactions can identify their roles in their families and community. Older adults derive their identity by belonging to a certain group or/and through their community roles. One older person described himself as a photography enthusiast who uses his laptop to store and share his photographs globally. Performing certain roles also gives older adults an identity in society. For example, they enjoyed providing caring support to their grandchildren when their children were at work or on vacation. One older adult explained how owning a car allows her to fulfil her role as a caregiver for her grandkids by making school drop- offs and pickups. Another older adult identified his role as a community gardener while describing the role of his smartphone in coordinating with other community gardeners.

*“… can take pictures of the garden, share updates online and coordinate with others on topics such as decisions on the produce.”* (Participant 12, male)

Seniors strive to preserve family traditions and rituals. Social values in older adults pertain to lifelong traditions that they have been following in their lives. As children move out of the house, some living interstate and overseas, it has become difficult for older adults to maintain these traditions. Maintaining traditions and rituals with family and friends also contributes to social values. One older adult stated that technology (Skype) allowed her to contact her extended family and friends every year during holidays, such as Thanksgiving and Christmas, as they live far away and cannot meet and celebrate together in person.

*“… used to meet over thanksgiving and Christmas…children have moved away, but they still call on those days on Skype.”* (Participant 26, female)

### 4.4. Association between the Themes

**Figure 4:**
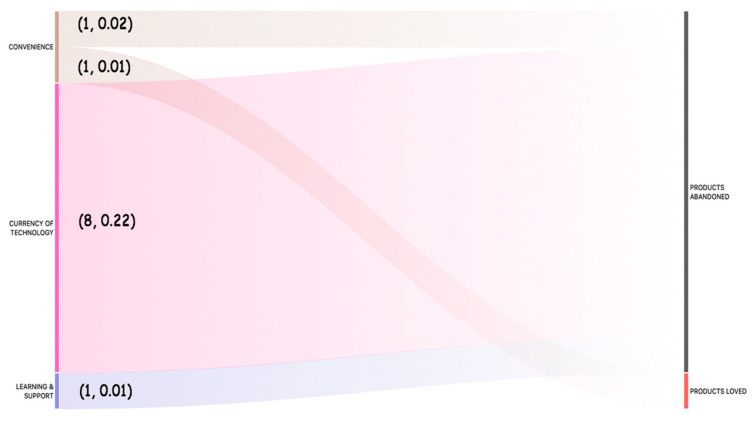
Sankey diagram showing relationship (co-occurrence) of “products loved” and “products abandoned” with the themes that represent how older adults value and experience technologies. (x, y) → x represents how often the two themes co-occur, y represents co-occurrence coefficient.

The co-occurrence between the coded themes was evaluated to identify relationship between them. The co-occurrence tool searches for themes that have been applied either to the same quotation or to overlapping quotations. This way, we could identify themes that are close to each other or mentioned together by the participants. We searched for all the co-occurrences using WITHIN, ENCLOSES, OVERLAPS, OVERLAPPED BY and the AND operators. A Sankey diagram in Figure 4 visualizes the association of the themes in the coded data. The numbers (x, y) indicate how often the two themes co-occur (x) and the co-occurrence co-efficient (y). The co-efficient indicates the strength of the relationship between the codes and ranges between 0 and 1.

#### 4.4.1. Association of “Products Loved” and “Products Abandoned” with the Themes *That Represent How Older Adults Value and Experience Technologies*

The results in Figure 4 indicate that the primary reason for products abandoned by older adults is that they own obsolete technologies. Themes ‘Products Abandoned’ and ‘Currency of Technology’ have co-occurrence count (8) and relationship strength (0.22).

#### 4.4.2. Association between Themes *That Represent How Older Adults Value and Experience Technologies*

**Figure 5:**
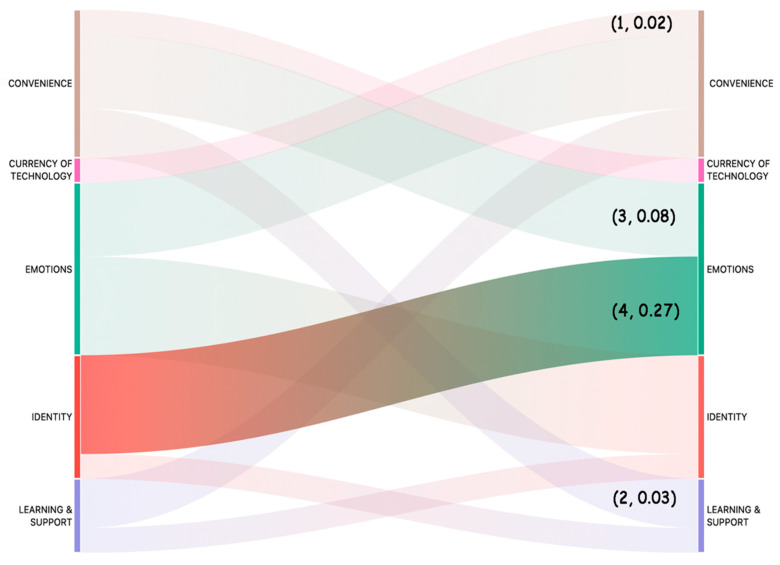
Sankey diagram showing relationship (co-occurrence) between themes representative of experiential value of technologies in older adults. (x, y) → x represents how often the two themes co-occur, y represents co-occurrence coefficient.

The results in Figure 5 indicate that the themes ‘Identity’ and ‘Emotions’ have maximum co-occurrence count (4) and relationship strength (0.27) followed by the themes ‘Convenience’ and ‘Emotions’ (3, 0.08), ‘Convenience’ and ‘Learning & Support’ (2, 0.03) and ‘Convenience’ and ‘Currency of Technology’ (1, 0.02).

#### 4.4.3. Association between Sub-Themes *of Convenience and Learning and Support*

**Figure 6:**
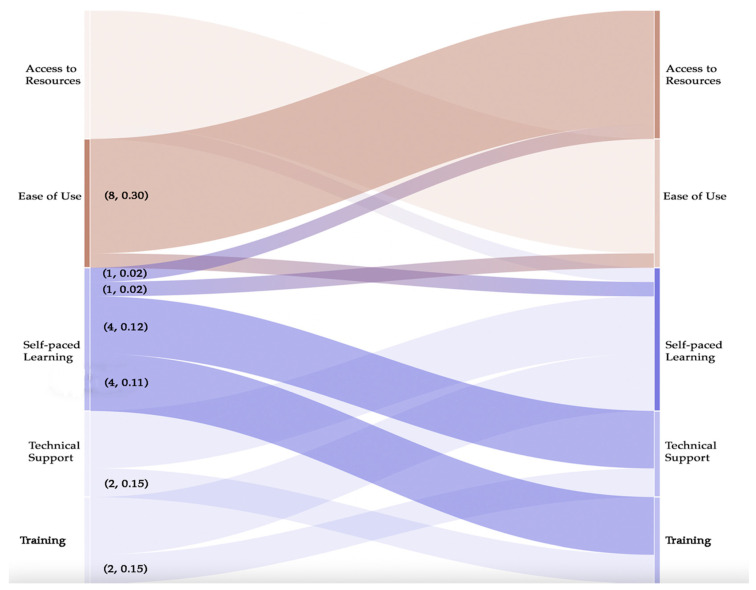
Sankey diagram showing relationship between sub-themes of Convenience and Learning & Support.

We found that the sub-themes ‘Ease of Use’ and ‘Access to Resources’ had a co-occurrence count of (8) and co-efficient of (0.30). ‘Self-paced learning’ is associated with ‘Technical Support’ and ‘Training’ with a count of (4) and co-efficient of (0.12) and (0.11) respectively. A Sankey visualisation of the associations is shown in Figure 6. 

## 5. Discussion

The study aimed to understand how older adults value technologies based on their past experiences with technologies that they have owned previously or own currently. This was done by bringing together seniors and other stakeholders (caregivers, technology developers, medical practitioners, and technology developers) and sharing their perspectives on technologies with each other. In the process, they co-created an experiential value framework for technology use in older adults.

Participants presented technologies (or their pictures) that they had brought along and explained why they loved or hated these technologies. A definition of technology was not provided to the participants as we also wanted to understand what technology meant to our participants. Older adults presented a range of technology products (Figure 1 and Figure 2). This included computers, tablets, smartphones, health management devices, cars, and cameras. The discussions between the participants at the round table suggests that older adults define technologies as products that make their everyday tasks easier. Some older adults expected technologies to be “automatic” in nature. Computers and smartphones were the most loved devices, while in-vehicle GPS and media players were the most hated technologies.

The thematic analysis found seven main factors that older adults value in their technologies. These values were driven by their experiences with these technologies and comprised of: Convenience, Economy, Learning and Support, Currency of Technology, Privacy and Security, Emotions and Identity. Boztepe conducted a study with users in Turkey to understand the cultural context of product use and identified utilitarian, emotional, and social values associated with product properties in the cultural contexts of product use such as treating guests, uses of space, and shopping [25]. Unlike Boztepe’s study that focused on use of products in Turkish kitchens, our study identified experiential factors associated with technology use in older adults over a period of time.

Previous studies examining older adults’ use of technologies were either carried out using technologies provided by the researchers or focused on specific assistive devices, such as telecare [36,37,38]. Limited studies have evaluated communication technologies, mobile phones and assistive technologies for older adults using the Technology Adoption Model (TAM) [39,40,41,42]. TAM was developed in studies completed without the involvement of technology consumers. The model also suggests that perceived usefulness and attitudes towards technology are contributing factors to technology adoption. However, TAM does not explain what influences usefulness and attitudes in the context of older adults, despite these factors depending on individual experiences. This study focused on technologies that older adults own and use in their everyday lives. The technologies were evaluated by the older adults based on their experiences with them over a period of time. This is the first known study where older adults co-create experience-based values using TUNGSTEN tools with caregivers, technology developers, and medical practitioners. Thematic analysis of the workshop data identified seven main themes representing experiential value in older adults: Convenience, Economy, Learning and Support, Currency of Technology, Privacy and Security, Emotions, and Identity. Some of these factors are unique to older adults, while some may also apply to younger populations.

The study identified ‘Learning and support’ as older adults’ most valued factor in the use of technologies (Figure 3). Further co-occurrence and association analysis suggests that available “Technical Support” and “Training” (sub-themes of learning and support theme) for technologies could allow older adults to follow self-paced self-learning of technologies. Ongoing technical support and training services not only instil confidence in older adults in the use of technologies but also prepare them to solve problems that arise with the technologies in the future. Most older adults in the study commented on the unavailability of instruction booklets and the poor quality of available instructions. Older adults prefer to use instruction manuals even before using a new technology, as the preference for trial and error decreases with age [43]. This preference could also be due to past negative experiences with technology. On the other hand, young adults are keen to explore and learn, and most often figure out how to use a technology without opening an instruction booklet. They are also much more technology savvy as compared to most seniors, as most are introduced to latest models of technology very young in their life.

Economy is the second most valued factor in the use of technologies. On average, older adults rely on their retirement savings for everyday expenses. Older adults, especially those who are socially and economically marginalized, cannot afford to invest in technologies like smartphones and laptops. Cost and affordability of technologies are critical for younger adults as well, especially for the successful adoption of communication technologies [44]. Ever-increasing phone bills put an additional financial strain on parents of teenagers [45]. During the COVID-19 pandemic, a lack of access to smartphones and computers has presented challenges to both age groups in accessing essential services [46].

The analysis revealed “Convenience” as the third most valued factor. Co-occurrence analysis of sub-themes of “Convenience” shows that access to resources contributes to the useability of technologies. This indicates that ease of use is not only about the way a product is designed but also about access to the infrastructure required to use that technology. Convenience of using a technology is equally important to younger adults. A study conducted by [47] to understand the role of technology in supporting older adults from the Jane and Finch community during the COVID-19 pandemic found that seniors did not have the required infrastructure and resources (such as WIFI and smartphones) to access seniors’ programs on Zoom. Younger adults in the community are also facing similar challenges.

The study also suggests the association of emotions and identity factors with technologies. A sense of identity within a family structure and community at large drives technology-related sentiments and emotions in older adults. This association is primarily due to activities that older adults carry out with family and friends which not only offers sentimental value but also gives them an identity in the community and at home. For example, doing school pickups and drop-offs for their grandchildren gives older adults a sense of identity in the family, an opportunity to socially interact with their loved ones, and tackle social isolation.

Most older adults at the workshop abandoned their technologies because they had become obsolete. “Currency of Technology” is associated with ‘Convenience’, primarily because modern technologies have features and properties that make use of technologies easy for older adults. Although keeping up with current technology is important for younger adults as well, it has not been difficult for them to utilize these technologies. According to Pew Research Centre, 96% of young adults aged 18–29 years own a smartphone compared to 60% of seniors in the USA [48].

Privacy and Security’ did not come out as a predominant factor that determines experiential value of technologies in older adults. However, older adults are very concerned about virus attacks and malwares that could steal their personal information and identity. This fear is due to increase in cases of fraud against seniors which is broadcasted in mainstream news and media channels [49]. Phone scams are the most common frauds used against seniors [50]. Seniors are made to believe that they owe money to the government or their family members are in danger and are asked to send money. Most seniors believe that Windows computers are more prone to phishing and malware attacks than Apple products. This is partly due to branding efforts of technology developers, but they are also influenced by their younger family members when it comes to choosing technology.

Based on the findings from this study, we recommend following design implications for developing technologies for older adults. These implications can be used as a guideline for developing accessible innovative person-centred solutions for older adults and other stakeholders.

### 5.1. Ecosystem of Technologies and Services

Use of technology requires a coordinated system of technologies, infrastructure, and services. Older adults require resources to learn and train to use technology. This includes instruction booklets with clear step-by-step instructions on how to set up, use, and adapt a technology to their own needs and requirements. There is a need to innovate the way instruction booklets and manuals are designed. For example, Lego moved away from text-based manuals and instead provides step-by-step visual directions in their instruction booklets. Services, such as the BILT app, provide 3D interactive instructions for a large range of products, from consumer-packaged goods to industrial and professional items. The BILT app also has an option to request instructions for a product which is not included in the app-based service. The service also has contact information for support, accessible at the tap of a button.

Older adults expressed the need to have an ongoing support for technologies they own. This support could include technical training during initial stages of procuring the technology and technical support over the period of using the technology. For example, older adults require support for connecting technology to other devices, using communication protocols (such as Bluetooth), changing device settings, and dealing with new firmware updates and errors (often shown as an error code) displayed by the technology.

Over the years, some organizations and technology developers have been offering courses and training on technologies for older adults. However, older adults (especially those who are not part of a program within these organizations) do not have access to this information. A portal service could provide an evolving list of training and courses offered for older adults to learn about technologies, books, tutorials, etc.

Some of these technologies require a well set up infrastructure, such as a high-speed internet connection and access to latest modern models of smartphones and computers. Services need to be developed to support older adults to gain access to this infrastructure. For example, the Toronto Public Library allows members to borrow tablets. However, more services need to be explored as an intervention to make every day and new technologies accessible to older adults.

Self-paced self-learning in older adults could be facilitated by ongoing technical support from the manufacturer. For example, Apple Care helpdesk support provides unlimited support for Apple products, ranging from setting up technologies to addressing malfunctions in the products. There are third party service providers, such as Seniors Tech Services, that provide in-home training and technical support to seniors. However, these are paid services and could be unaffordable for the majority of the older population.

### 5.2. Support for Privacy and Security in Technologies

Most technologies require us to share our personal information, such demographics and health data. Older adults expressed concern over sharing their private and personal information. One of the reasons for this concern is the lack of trust in governing and private organizations. Older adults are concerned about how their information will be used by these organizations. For example, the Quantas wellbeing app (a companion app that goes hand-in-hand with Quantas insurance products) tracks a user’s health (sleep, blood pressure, etc.), eating habits, and exercise routine. The premiums on the insurance products vary based on the recorded data [51].

Cybersecurity is a major concern for older adults. There is fear that their technologies could be infected by malware and viruses, and their identity and personal information could be hacked [52]. Older adults who do not have a technical background or experience could find technical terminologies related to viruses difficult to understand [53]. This makes it difficult for them to invest in an appropriate anti-virus software. Those who have anti-virus software installed on their technologies find it difficult to understand the notifications, warnings, and messages [52,54]. Thus, there is a need to design anti-virus and anti-phishing software that is easy to install, update and understand. The language used in these software programs should be simple and without technical jargon.

There are certain perceptions and assumptions that seniors have developed on topics related to privacy and security. For example, a study on technology use with older adults from the Jane and Finch community during the COVID-19 pandemic found that seniors prefer to use WhatsApp because it is more secured and safer than Facebook. Seniors who use Facebook do not understand how it works, often resulting in misunderstandings during social interactions. Facebook disrupts the way we normally socialize in the physical world. This disruption is inconvenient and renders the platform unusable for many seniors. Thus, we recommend that there is a need to generate awareness about privacy and security amongst seniors. This could be done through various mediums, such as information pamphlets at day programs, product manuals or information sessions. The information could be integrated into existing programs and resources. For example, the Royal Canadian Mounted Police (RCMP) has an online resource called “Seniors Guidebook to Safety and Security” [55]. The Canadian federal government has also created an online resource for elderly entitled “What every older Canadian should know about” [56]. However, this document has not been updated since 2010. These resources do not include facts and information on privacy and security. These resources also lack information on how to safeguard oneself while using technology. 

### 5.3. Technology as a Tool to Help Seniors Build an Identity

Perceived ease of use was identified as a key determinant of technology adoption in TAM model [42]. Older adults in this study related the usefulness of the technology to the activities that they can carry out with the technology. They reported that technologies that they use regularly make everyday tasks easy. For example, seniors used technologies to make phone calls, send emails, take photographs, share photos with friends and family, stay in touch with loved ones, and track their health and wellbeing. Having fun doing these activities and enjoying their interactions with the technology is important for emotional wellbeing of older adults [57,58]. When these activities are done with loved ones, it adds sentimental value to the use of the technology.

Older adults strive for identity within their communities and families. This sense of identity is derived from playing a role in the lives of loved ones, for example acting as care givers to their grandchildren. Older adults consider technology helpful in achieving their goals and objectives. Older adults value the contribution of technology in maintaining traditions and rituals. For example, technology helps them keep in contact with family and friends at annual holidays. It is our recommendation that technologies be developed to meet specific needs for older adults rather than as tools that automate tasks for them. Technologies should be designed to embody activities and rituals that seniors engage in [59]. This could mean that technologies could facilitate storytelling sessions with grandchildren, support social interactions with friends or help seniors indulge in a hobby. Makers Making Change in Toronto, Canada, is an organization that leverages the ability of community-based makers, occupational therapists, and volunteers to develop and deliver affordable, open-source assistive technologies. Some seniors are participating in similar organizations to contribute to their communities while indulging in a hobby.

### 5.4. Affordability

Although technologies are highly valued based on the activities afforded and services available to support them, the cost of technologies is a major factor that could drive older adults away from them [60]. The cost of technologies could also be the reason that older adults rely on used technologies, previously owned by their children and grandchildren. Heinz and others found in their study that older adults feel apprehensive of the cost of new technologies; however, their benefits outweigh the cost [61]. Steele on the other hand found that cost played a major role in older adults adoption of wireless sensors to assist in their healthcare management and tracking [62]. This discrepancy could be due to motivation and need to use certain technologies. Our study found that older adults were concerned about the ongoing expenses that they must pay to use the technology on top of its initial cost. For example, Muse, an EEG-powered sleep tracking and meditation device, costs between $300 and $500 depending on various features and tracking abilities. The real tracking power comes from the ongoing use of an app to track one’s wellbeing, which costs around $130 per year. Older adults who do not see the immediate need and benefit of the product will not adopt the technology [3]. As one of the older adults at the workshop suggested, there should be government policies around subsidies and incentives around accessing certain technologies. This could become very beneficial for older adults living in marginalized communities [63].

In summary, technologies for seniors should meet following criteria for ongoing use and adoption of technology in everyday life:

1. Designers and technology developers should strive to build product ecosystems of technologies, resources, services, and infrastructure. Innovations in design of instruction manuals is required.
2. Attempts to create awareness and information on privacy and security issues with all technical jargon clearly explained either visually or in text.
3. Anti-virus and anti-phishing software need to be accessible to seniors. They should be easy to download and install updates. Notifications should be easy to understand without any use of technical jargon.
4. Technology should be designed and developed to meet needs of older adults to create an identity for themselves in the family and community.

## 6. Conclusions

This study has focussed on understanding what older adults value in technologies based on their experience with technologies that they own. The findings suggest that older adults value Convenience, Economy, Learning and Support, Currency of Technology, Privacy and Security, Emotions, and Identity aspects of their experiences with their technologies. The study has resulted in implications to design that recommends (a) Ecosystem of technologies and services: design for product ecosystems with technologies and services well-coordinated to meet needs for resources to make use of technologies easy (b) Privacy and Security: create awareness and information on privacy and security issues while using technology (c) Easy to use anti-virus and anti-phishing software and make these software accessible to older population (d) Identity: design technologies as tools that allow older adults to identify themselves in the community and family (e) Affordability: Services that make technologies and services in the ecosystem affordable for the older adults. The outcomes of this study are significant as they provide recommendations that target systemic issues that act as barriers in the use of technologies for older adults. This is contrary to previous studies that have focussed on usability issues with the technology itself.

## Figures and Tables

**Table 1:** Themes and sub-themes representing how older adults value everyday technologies based on their experiences with them.

Themes	Sub-Themes	Quote
Convenience	Ease of Use	“My new tablet allows me to skype anywhere in my house.” “… [it] can work [remotely] via email.” “E-reader is good because you don’t need to carry books around…” “Smartphone talks to car, can pick up while driving.”
	Access to resources	“I can call and text with my smartphone, but it has limited amount of data.”
Economy	-	“… [Laptop]] was on sale…” “… Upgraded to new [phone] as it was offered by Rogers [telecoms provider] on a new phone plan for no charge.”
Learning & Support	Instruction booklet	“… [I] tried to figure out [how to transfer photos from my Kodak Easy Share photo frame] with instructions in the box but was unsuccessful…”
	Self-paced learning	“I self-taught myself to use my smart phone.” “I learnt to use the Kindle myself.” “I like to trial error and learn how to use… it may not work at times… I like to be independent of my kids.”
	Technical support	“My Mac laptop had minimal technical support … I kept running into problems and there was no help.” “My daughter helps me now and then with my IPad mini.”
	Training	“… [I] took public classes to learn how to use Facebook” “I went to IPhone school at the mall.”
Currency of technology	-	“Landline does not have as many functions [as smartphones].” “I don’t like my DVD player because there are new [technologies] out like Blue Ray and Netflix.”
Privacy and Security	-	“[I love my smartphone because] it has great security features.” “[My Macbook pro] is safe as it removes all viruses every day.” “[My Windows computer] has malware pop ups and viruses”
Emotions	-	“The necklace [wearable technology in the form of a necklace] is not my style. It does not look good on me.” “… [I] love my iPad. I have fun playing games on it.”
Identity	-	“[Laptop] can save lots of pictures, allows to edit them and share it on my blog and social media.”
		“… used to meet over thanksgiving and Christmas…children have moved away, but they still call on those days on Skype.”

## Data Availability

The audio and video data identify participants and their personal information. Thus, the data presented in this study are not publicly available due to privacy and ethical restrictions imposed by the Research Ethics Boards.
